# Biomapping of Microbial Indicators on Beef Subprimals Subjected to Spray or Dry Chilling over Prolonged Refrigerated Storage

**DOI:** 10.3390/foods10061403

**Published:** 2021-06-17

**Authors:** Diego E. Casas, Rosine Manishimwe, Savannah J. Forgey, Keelyn E. Hanlon, Markus F. Miller, Mindy M. Brashears, Marcos X. Sanchez-Plata

**Affiliations:** International Center for Food Industry Excellence, Department of Animal and Food Sciences, Texas Tech University, Lubbock, TX 79409, USA; diego.casas@ttu.edu (D.E.C.); r.manishimwe@ur.ac.rw (R.M.); savannah.forgey@ttu.edu (S.J.F.); keelyn.hanlon@ttu.edu (K.E.H.); mfmrraider@aol.com (M.F.M.); mindy.brashears@ttu.edu (M.M.B.)

**Keywords:** refrigerated meat shelf life, microbial indicators, vacuum packaging, carcass chilling, hot water intervention

## Abstract

As the global meat market moves to never frozen alternatives, meat processors seek opportunities for increasing the shelf life of fresh meats by combinations of proper cold chain management, barrier technologies, and antimicrobial interventions. The objective of this study was to determine the impact of spray and dry chilling combined with hot water carcass treatments on the levels of microbial indicator organisms during the long-term refrigerated storage of beef cuts. Samples were taken using EZ-Reach™ sponge samplers with 25 mL buffered peptone water over a 100 cm^2^ area of the striploin. Sample collection was conducted before the hot carcass wash, after wash, and after the 24 h carcass chilling. Chilled striploins were cut into four sections, individually vacuum packaged, and stored to be sampled at 0, 45, 70, and 135 days (*n* = 200) of refrigerated storage and distribution. Aerobic plate counts, enterobacteria, *Escherichia coli*, coliforms, and psychrotroph counts were evaluated for each sample. Not enough evidence (*p* > 0.05) was found indicating the hot water wash intervention reduced bacterial concentration on the carcass surface. *E. coli* was below detection limits (<0.25 CFU/cm^2^) in most of the samples taken. No significant difference (*p* > 0.05) was found between coliform counts throughout the sampling dates. Feed type did not seem to influence the (*p* > 0.25) microbial load of the treatments. Even though no immediate effect was seen when comparing spray or dry chilling of the samples at day 0, as the product aged, a significantly lower (*p* < 0.05) concentration of aerobic and psychrotrophic organisms in dry-chilled samples could be observed when compared to their spray-chilled counterparts. Data collected can be used to select alternative chilling systems to maximize shelf life in vacuum packaged beef kept over prolonged storage periods.

## 1. Introduction

The world beef market is heavily influenced by consumer demands and choices; therefore, the beef industry must adapt to the consumers’ needs and concerns and provide meat products that fulfill such needs. Certain consumer demands have created niche opportunities for a variety of meat product offerings. An important market niche for beef products is the “fresh meat” “never frozen” alternatives. This has led meat processors to seek schemes for increasing the shelf life of fresh meats by combinations of proper cold management, barrier technologies, and application of antimicrobial interventions (chemical or physical) [1,2]. The growing demand for fresh products has put pressure on the cold supply chain and quality control at all steps in the processing plant [3]. Such a trend has evolved rapidly, and now regulatory agencies have developed a series of labeling requirements for never frozen meat and poultry products. The U.S. Department of Agriculture Food Safety and Inspection Service (USDA-FSIS) has defined that any poultry product below −3.3 °C (26 °F) or red meat that has ever been frozen cannot be labeled as fresh, not frozen [4]. To address these market trends, beef processors need to explore novel processing schemes, product protection options, and process modifications that have been properly validated in commercial settings to extend product shelf life, especially when long transport regimes are necessary under refrigerated conditions due to significant distances between production and market locations.

Australia is one of the leading beef exporters in the world. As an important market player, the Australian beef industry has been continuously assessing new market opportunities and has been exploring fresh, never frozen beef alternatives for competitive markets worldwide. In 2018, Australia’s bovine meat exports accounted for 43.2% of animal product exports with a market value of approximately $6.47 billion for the country’s economy. Australia’s biggest beef export market is Japan with a market share of 36.8% in 2018. With the recent interest of the United States to significantly increase its beef exports to Japan [5,6], Australia has sought opportunities to expand its presence in the European Union. This high-income market shows significant consumer interest in the fresh, never frozen beef products [7]. Unfortunately, the distance between the meat source and the EU market has created a challenge, due to the long-haul transportation needs and rigorous chilled conditions necessary for product arrival and suitability for fresh distribution. Consequently, extending the shelf life of chilled meat products has become of the utmost interest.

Meat shelf life extension has been achieved through the use of several antimicrobial interventions, chilling methods, and barrier technologies in the past [1,2,8,9]. At the same time, these interventions and barrier technologies mitigate the growth of indicator and pathogenic bacteria that are responsible for product deterioration. The hot water wash of carcasses has been observed to reduce 2.7–3.0 log CFU/cm^2^ of *L. monocytogenes*, *Salmonella*, and APC counts [10]. Dry aging has been observed to reduce over 2 log CFU/cm^2^ of generic *E. coli* and *E. coli* O157:H7 in beef carcasses and subprimals [11,12]. Air chilling has been shown to reduce total viable counts by 03–0.7 log CFU/cm^2^ [13], and up to 2 log CFU/cm^2^ of *E. coli* [14]. Moreover, indicator and pathogenic microorganisms have been reduced after the air conventional chilling and blast chilling of carcasses [15,16]. Comparatively, spray chilling has been observed to have no immediate effect in microbial populations [17]. Thus, in this study, we evaluate the use of hot carcass washing and different carcass chilling systems to assess Australian chilled beef’s extended shelf life in export settings that require product viability for more than 130 days of refrigerated storage and distribution.

## 2. Materials and Methods

### 2.1. Sample Collection

Samples were taken at Teys Australia beef processing plant located in Beenleigh, QLD, Australia. A total of 200 carcasses were evaluated. Swab samples were taken using EZ-Reach™ Sponge Samplers hydrated with 25 mL buffered peptone water (BPW, World BioProducts, Mundelein, Illinois) by swabbing an over 100 cm^2^ area on the striploin region of each carcass. Samples were taken before the hot carcass wash, after the hot carcass wash for washed samples, and 24 h after being subjected to one of the chilling methods described below (spray vs. dry chilling). Edible ink was used to mark the area where the sample was taken to avoid re-sampling of the same surface. The hot water carcass wash consisted of spraying 85 ± 2 °C water onto the surface of the carcass through eight nozzle sprayers, four per side of the carcass. Water temperature was recorded on the pipes feeding the water to the sprayers right before sample collection. The chilling methods evaluated consisted of 18–24 h storage in a refrigerated chamber subjected to continuous spraying of water at 0–2 °C in the room at 15 min intervals, following the processing plant’s protocols. Dry chilling consisted of 18–24 h storage in a refrigerated room at 0 °C with constant airflow while the sprayers were completely turned off. After 24 h chilling, either under water spray conditions or dry refrigerated storage, striploins were taken and cut into 4 sections. Individual sections were vacuum packaged and assigned a date for further sampling at 0, 45, 70, and 135 days of refrigerated storage. Samples collected for day 0 were analyzed in an in-plant laboratory setup at the processing facility. Striploins were shipped via sea to the ICFIE Food Microbiology Laboratory at Texas Tech University (TTU) in Lubbock, Texas, USA for the long-term shelf life section of the study corresponding to storage at days 45, 70, and 135 under refrigerated conditions. Striploins were kept at 0–4 °C from carcass fabrication to meat reception at TTU. On day 40, striploins were received at TTU and the refrigerated temperature was raised to 7 °C, simulating abusive counter temperatures common in retail stores. On each sampling day, striploin packages were opened with sterile scalpels and an area of 100 cm^2^ of the product was swabbed for sample collection.

### 2.2. Sample Processing

Swab samples collected were stomached for 30 s at 230 rpm. Serial dilutions for each swab sample were made with 9 mL BPW tubes. A volume of 1 mL was plated onto Petrifilm™ plates (3M, Saint Paul, Minnesota) in duplicate corresponding to *Enterobacteriaceae* (EB), *Escherichia coli* (EC), coliforms (CO), and aerobic plate counts (APC). In addition, aerobic plate count Petrifilm was also used to estimate psychrotroph counts (PSY) by incubating separate plates at 20 °C for 72 h [18,19]. *Enterobacteriaceae* Petrifilms were incubated for 24 h at 37 °C before counting. Coliforms were counted after 24 h incubation at 37 °C. *Escherichia coli* counts were recorded after 48 h incubation at 37 °C following the manufacturer’s recommendations. APC plates were incubated for 48 h at 37 °C.

### 2.3. Experimental Design and Statistical Analysis

The hot water wash section of the study had a completely randomized design with a factorial arrangement of 2 factors, feed regime and carcass wash, at 2 levels each: grass vs. grain and washed vs. not washed, respectively. Three sampling points were evaluated, before wash, after the washing stage, and after a 24 h chilling period. For each repetition, 10 samples were taken per treatment (Table S1) at each sampling point. A total of 5 repetitions were conducted.

The section of the study regarding the extended shelf life of the striploins was arranged in a completely randomized design with a factorial arrangement of three factors, feed regime, hot water wash application, and chilling method, at two levels each: grass vs. grain, washed vs. not-washed, and dry vs. spray-chilled, respectively. For each repetition, 5 samples per treatment were taken at each sampling date (Table S2). A total of 5 repetitions were conducted resulting in 200 samples per sampling date. An ANOVA by sampling date was used to analyze the data when parametric assumptions were satisfied. The Kruskal–Wallis (nonparametric ANOVA) test was used to analyze the data when parametric assumptions were not met. When the ANOVA or Kruskal–Wallis was significant, pairwise comparisons were done using a pairwise T-test on significant ANOVAs or a Wilcoxon rank-sum test on significant Kruskal–Wallis tests [20]. Statistical significance was evaluated at a 0.05 probability level.

## 3. Results

### 3.1. Hot Water Wash

**Figure 1:**
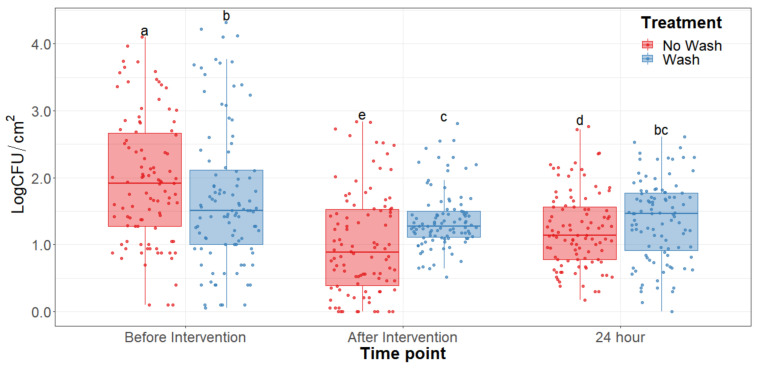
Aerobic plate counts of the beef carcass surface before and after the hot water wash intervention and 24 h chilling period. The horizontal line within the box plot represents the median. The box upper and lower limits represent the interquartile range, and the bars represent the 1.5xInterquartile Range. ^a–e^ Box plots with different letters are significantly different (*p* < 0.05).

**Figure 2:**
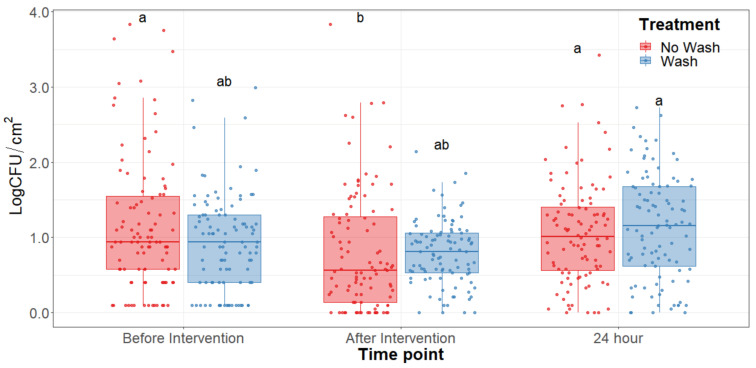
Psychrotroph counts of the beef carcass surface before and after the hot water wash intervention and 24 h chilling period. The horizontal line within the box plot represents the median. The box upper and lower limits represent the interquartile range, and the bars represent the 1.5xInterquartile Range. ^a,b^ Box plots with different letters are significantly different (*p* < 0.05).

The main effect of the feed type had no statistical significance throughout any of the sampling dates of the study. There was no significant difference (*p* > 0.25) on the bacterial counts observed between grain and grass-fed carcasses in the study; therefore, the main effect of the feed type was removed to better visualize differences due to the washing and chilling types’ main effects and their interaction. The hot water wash carcass intervention significantly reduced (*p* < 0.05) APC on the carcass surface (Figure 1). However, no washed treatments presented lower aerobic plate counts than the washed counterparts. After a 24 h chilling period, there was an increase in PSY counts and a stalled growth of APC. Psychrotrophic bacteria were not significantly reduced by the hot water wash intervention (Figure 2) and had growth after a 24 h chilling period. EB, EC, and CO counts were below the detection limit (<0.25 CFU/cm^2^) in most samples taken at each sampling point assessed.

### 3.2. Extended Shelf Life of Striploins

**Figure 3:**
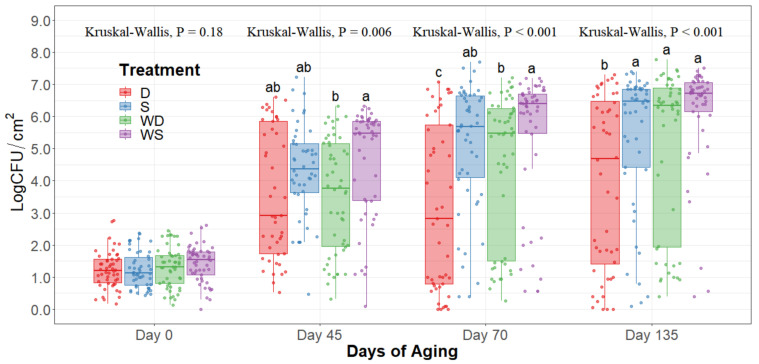
Aerobic plate counts of striploins at day 0, 45, 70, and 135 of refrigerated storage. The horizontal line within the box plot represents the median. The box upper and lower limits represent the interquartile range, and the bars represent the 1.5xInterquartile Range. D = No Wash Dry chill, S = No Wash Spray chill, WD = Wash Dry chill, WS = Wash Spray chill. ^a–c^ Box plots with different letters within each sampling date are significantly different (*p* < 0.05).

**Figure 4:**
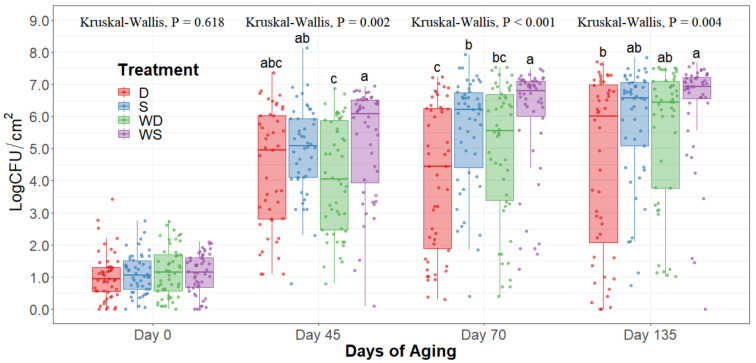
Psychrotroph counts of striploins at day 0, 45, 70, and 135 of refrigerated storage. The horizontal line within the box plot represents the median. The box upper and lower limits represent the interquartile range, and the bars represent the 1.5xInterquartile Range. D = No Wash Dry chill, S = No Wash Spray chill, WD = Wash Dry chill, WS = Wash Spray chill. ^a–c^ Box plots with different letters within each sampling date are significantly different (*p* < 0.05).

**Figure 5:**
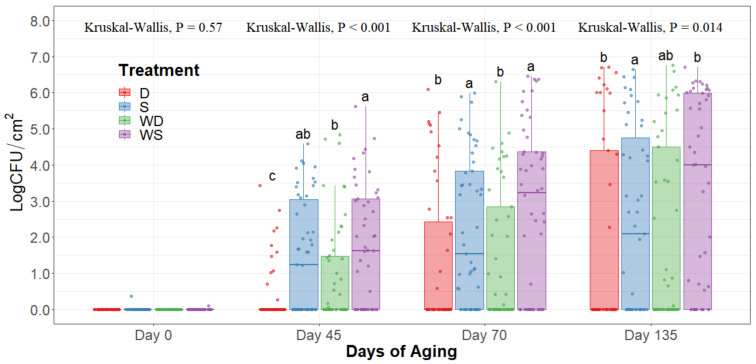
Enterobacteriaceae counts of striploins at day 0, 45, 70, and 135 of refrigerated storage. The horizontal line within the box plot represents the median. The box upper and lower limits represent the interquartile range, and the bars represent the 1.5xInterquartile Range. D = No Wash Dry chill, S = No Wash Spray chill, WD = Wash Dry chill, WS = Wash Spray chill. ^a–c^ Box plots with different letters within each sampling date are significantly different (*p* < 0.05).

The statistical analysis indicates a significant effect of time for all the indicator microorganism loads assessed, as expected. Because of this, the statistical comparison between treatments was conducted within a per sampling date basis, rather than over the time of storage. The loads of each indicator microorganism evaluated was compared between treatments within each sampling date. On sampling day 0, no significant differences among treatments could be observed in any of the five microbial indicators quantified (*p* > 0.05) and the indicator bacteria were mostly below the detection limit (Table 1). Even though no immediate effect could be observed from spray and dry chilling at day 0, in the long term, and throughout the additional sampling periods during refrigerated storage, significantly lower (*p* < 0.05) concentrations of APC, PSY, and EB can be observed in the dry chilling treatments (Figure 3, Figure 4 and Figure 5) when compared to their spray-chilled counterparts.

No significant differences on coliform counts between treatments at each sampling date could be found throughout the extended shelf life section of the study; however, significant growth over time was observed. *E. coli* counts on striploins were mostly below the detection limit (<0.25 CFU/cm^2^) at the plant and throughout the extended shelf life. Thus, no significant growth of *E. coli* over time was observed. Furthermore, significant growth of EB was observed only after 45 days of wet aging, encountering significant differences between treatments during long-term storage.

Even though the hot water wash’s main effect was not statistically significant throughout the extended shelf life study, a trend (0.05 < *p* < 0.15) of an increase in microbes quantified could be observed whenever the carcasses underwent the hot water wash intervention compared to their dry chilling counterparts. The highest microbial concentrations were consistently observed on the washed and spray-chilled striploins treatment and the lowest microbial loads were consistently observed in the no-washed dry-chilled striploins. Although significant interaction between the main effects was not observed statistically, a trend in the interaction was observed (0.05 < *p* < 0.15).

EB counts were significantly different between treatments after long-term storage (*p* < 0.05). Dry chilling methods had their medians at 0 log CFU/cm^2^, indicating a low concentration of EB even after 135 days of refrigerated storage. Moreover, the no-wash dry chilling treatment combination had the lowest concentration of EB across all times evaluated. The treatment’s significant differences after prolonged refrigeration times become evident from day 45 of long-term refrigeration storage.

## 4. Discussion

Microbial indicator levels assessed in the hot carcass wash section of the study were substantially lower before the hot carcass wash intervention, demonstrating the efficacy of proper sanitary dressing procedures in the facility (Table 1). Since the initial concentration of microorganisms was so low, no significant reductions in indicator bacteria concentrations were observed in the early stages of sampling and no effects were observed after subjection to the different treatment combinations. Because of this, the concentration of *Enterobacteriaceae, E. coli*, and coliforms were below detection limits (<0.25 CFU/cm^2^) after the hot water wash intervention in most samples collected at day 0. Under the conditions evaluated in this study, the hot water carcass intervention was not found to significantly reduce APC and PSY counts compared to no-wash treatments. This finding shows that despite significantly reducing a small number of bacteria on the surface of the carcass, washing the carcass may also redistribute the bacteria throughout the whole carcass surface and that can contribute to further differences during prolonged storage. This may pose a counterproductive result as bacteria will have a greater surface area of contact with the carcass and these may allow for more microbial attachment, growth, and development [21]. Furthermore, the washing of the carcass surfaces may increase available water for microbial growth which in the long term may allow a higher proliferation of bacteria in the striploins, a tendency observed in the long-term storage under refrigerating conditions [22]. Higher reductions may be achieved with alternative physical interventions such as steam vacuuming and trimming which do not use any chemicals for the reduction of bacteria [23,24].

When observing the hot carcass washing and type of carcass chilling, an immediate effect was not observed in any of the five bacteria quantified at day 0. Particularly EB, CO, and EC were all below detection limits within the in-plant sampling at day 0. By the time striploins had undergone shipment and distribution to export markets under refrigeration (day 45), a significant difference was observed between treatments in PSY, APC, and EB, where dry chilling treatments had lower bacterial counts overall. This trend was kept throughout the 135 days of refrigerated storage evaluated in this study.

APC evaluations show an overall count of mesophilic bacteria demonstrating a general picture of the total bacteria counts within the striploins. However, psychrotroph counts represent a more accurate bacterial load of meat, as meat is mostly stored under refrigerated conditions for a prolonged time. Previous research has demonstrated around a 0.5–1.0 log CFU increase in concentration on PSY counts compared to APC [18,25]. Most psychrotrophic enumeration methods require incubation at 7 °C for 10 days, or at 10 °C for 7 days, among others [19]. Furthermore, methods with incubation at 20 °C for 72 h have been used to enumerate carcass and meat subprimal psychrotrophic counts [18,26]. Due to variability in protocols for quantification of psychrotrophic bacteria counts, a trial comparing psychrotrophic counts using incubation at 7 °C for 10 days and 20 °C for 72 h was performed. Results led to a correlation of 0.96 and 0.98 at 45 and 70 days of aging, respectively (data not shown), thus validating the use of the protocol of incubation for psychrotrophic bacterial counts at 20 °C for 72 h. PSY counts were significantly lower on the dry chilling treatments, particularly in the no-washed dry-chilled treatment combination. Furthermore, package bloating and off-odors were less frequently found in striploins subjected to the dry-chilled treatment.

During long-term refrigerated storage of meat, Pseudomonas, Enterobacteria, and lactic acid bacteria become the main microorganisms that cause spoilage [27]. In the past, EB has also been used as an indicator of the risk of *Salmonella* spp. contamination. A higher concentration of EB may increase the risk of *Salmonella* spp. presence [28]. However, EB presence does not confirm *Salmonella* spp. presence. Similarly, pathogens of public health interest are within the EB family classification, such as *Shigella*, *E. coli*, and *Klebsiella*. In this context, all treatments were effective at mitigating EB presence at day 0 of storage; however, as the long-term storage continued, evident differences between treatments were observed, where dry chilling treatments more effectively mitigated EB proliferation in the striploins. This mitigation of EB growth throughout time may suggest that dry chilling not only prolongs shelf life but also further ensures food safety through lower rates of bacterial growth with the potential of increasing bacterial injury [8,29,30].

No difference between treatments was observed in CO and EC due to the low concentration encountered within sampling dates throughout the trial. EB and EC counts serve as a Gram-negative indicator of fecal contamination. Generic *E. coli* serves as an indicator of process control; the FSIS has published minimal sampling requirements for beef processors indicating that final carcasses must have negative results of *E. coli* to be considered acceptable; moreover, if more than four samples are between 1–100 CFU/cm^2^ in a window of 13 consecutive samples or a sample is over 100 CFU/cm^2^ a corrective action is warranted [31]. In this context, the beef processing plant is well within the acceptable limits of *E. coli* enumeration, having over 95% of the carcasses below the detection limit and all below 100 CFU/cm^2^ after harvest and throughout the long-term storage of striploins under refrigerated conditions. This is an outstanding indicator of proper hygiene procedures and sanitary dressing procedures within the plant. Previous research effectively validated process controls within beef slaughter operations using EC as an indicator of process control alongside APC and EB counts [26,32].

Overall, dry chilling procedures prolonged the shelf life of striploins more effectively. Dry aging has been observed to reduce *E. coli* O157:H7 and generic *E. coli* concentrations in carcasses [11,12]. Moreover, air chilling and blast chilling of carcasses have both shown similar results in the reduction of indicator microorganisms in beef and pork carcasses [15,16]. Contrastingly, spray-chilled carcasses have been shown to have no immediate effect on the microbial load of carcasses [17,30], as observed in this study. However, in the long term, spray-washed treatments consistently had higher microbial loads throughout all the treatments and no-washed dry-chilled treatments had consistently significantly lower microbial concentrations, suggesting different slopes for growth curves of microorganisms under different treatments. Importantly, dry chilling procedures are known to reduce cold carcass weight due to the loss of moisture from the carcass surface during chilling, resulting in economic loss for the processing plant [33,34]. The optimization of interventions, chilling techniques, and barrier technologies depending on the end consumer and shelf life requirements of the meat can result in minimization of economic loss, a better microbial quality, and a safer meat product.

## 5. Conclusions

A hot water wash prior to carcass chilling did not significantly reduce microorganisms assessed under the conditions evaluated in this study. Dry chilling of carcasses can potentially increase the shelf life of meat products as it delays the growth of bacteria under the refrigerated conditions of storage during transport and distribution. Data collected can be used to select chilling systems to maximize shelf life, especially in long-term refrigerated storage conditions of never frozen beef products. The optimal shelf life of striploins can be achieved using dry chilling air systems, which will guarantee the required 130 days of shelf life for the export of fresh, never frozen beef from Australia to the EU. The use of spray chilling schemes increases available water for the growth of bacteria resulting in higher growth rates of bacteria during the long-term refrigerated storage and therefore a reduced shelf life. This extended quality preservation over an extended shelf life period allows more flexibility in beef exports, especially for major producers that are far from target consumer markets. Understanding the best parameters for beef carcass processing and storage will allow the beef industry to select optimized chilling schemes for long-term storage and increased consumer acceptability.

## Figures and Tables

**Table 1:** Summary table of microbial indicator microorganism counts in striploins before and after the intervention, chilling method, and evaluation at day 0, 45, 70, and 135 of refrigerated storage.

Microorganism	Treatment	Timepoint (LogCFU/cm^2^ ± S.E. ^1^)					
		Before Wash	After Wash	Day 0	Day 45	Day 70	Day 135
Aerobic plate count	No wash Dry	1.96 ± 0.10	1.01 ± 0.04	1.23 ± 0.08	3.62 ± 0.29	3.19 ± 0.36	3.97 ± 0.38
	No wash Spray			1.23 ± 0.08	4.33 ± 0.20	5.1 ± 0.28	5.39 ± 0.31
	Wash Dry	1.72 ± 0.09	1.38 ± 0.08	1.31 ± 0.09	3.55 ± 0.25	4.51 ± 0.32	5.05 ± 0.35
	Wash Spray			1.42 ± 0.08	4.67 ± 0.24	5.41 ± 0.24	6.11 ± 0.24
Psychrotroph count	No wash Dry	1.16 ± 0.06	0.80 ± 0.04	1.02 ± 0.10	4.37 ± 0.27	4.09 ± 0.33	4.55 ± 0.38
	No wash Spray			1.10 ± 0.09	4.97 ± 0.20	5.54 ± 0.25	5.80 ± 0.26
	Wash Dry	0.93 ± 0.09	0.80 ± 0.08	1.17 ± 0.11	4.11 ± 0.24	4.96 ± 0.20	5.52 ± 0.30
	Wash Spray			1.11 ± 0.09	5.22 ± 0.24	6.00 ± 0.25	6.34 ± 0.23
Enterobacteriaceae count	No wash Dry	0.19 ± 0.05	0.04 ± 0.02	0.00 *	0.4 ± 0.12	1.23 ± 0.28	1.81 ± 0.38
	No wash Spray			0.01 ± 0.01	1.44 ± 0.22	2.18 ± 0.29	2.42 ± 0.35
	Wash Dry	0.02 ± 0.01	0.00 *	0.00 *	0.94 ± 0.20	1.39 ± 0.27	1.99 ± 0.35
	Wash Spray			0.00 *	1.71 ± 0.24	2.78 ± 0.33	3.28 ± 0.38
Coliform count	No wash Dry	0.02 ± 0.01	0.02 ± 0.01	0.12 ± 0.06	0.12 ± 0.08	0.22 ± 0.13	0.51 ± 0.24
	No wash Spray			0.19 ± 0.07	0.47 ± 0.07	0.47 ± 0.16	0.69 ± 0.25
	Wash Dry	0.00 *	0.00 *	0.04 ± 0.04	0.04 ± 0.04	0.24 ± 0.16	0.53 ± 0.23
	Wash Spray			0.17 ± 0.07	0.35 ± 0.07	0.66 ± 0.24	0.90 ± 0.29

^1^ Standard Error. * Below detection limit (<0.25 CFU/cm^2^).

## Data Availability

All data from the research conducted are available on request from the corresponding author. The data are not publicly available due to the privacy of our industry collaborator that allowed for the project to be conducted.

## Supplementary Materials

The following are available online at https://www.mdpi.com/article/10.3390/foods10061403/s1. Table S1. Experimental design of no washed and hot water washed carcasses in a beef processing facility at each sampling point, before and after carcass wash, and 24-h carcass chilling, Table S2. Experimental design at each sampling date for the extended shelf-life evaluation of beef striploins. Table S1. Experimental design of no washed and hot water washed carcasses in a beef processing facility at each sampling point, before and after carcass wash, and 24-h carcass chilling. Table S2. Experimental design at each sampling date for the extended shelf life evaluation of beef striploins.
