# Supplementary material for: Biomapping of Microbial Indicators on Beef Subprimals Subjected to Spray or Dry Chilling over Prolonged Refrigerated Storage
Source: Foods. 2021 Jun 17;10(6):1403. doi: 10.3390/foods10061403 (PMC8234038; doi:10.3390/foods10061403)
Supplement: Supplementary file 1 [file foods-10-01403-s001.zip › foods-1230180-supplementary.pdf]

Supplementary Table S1. Experimental design of no washed and hot water washed carcasses in a beef processing facility at each sampling point, before and after carcass wash, and 24-hour carcass chilling.

Grass Fed		Grain Fed	
Hot Water Washed	No Water Wash	Hot water Washed	No Water Wash
Treatment 1<br>N=50	Treatment 2<br>N=50	Treatment 3<br>N=50	Treatment 4<br>N=50

Supplementary Table S2. Experimental design at each sampling date for the extended shelf life evaluation of beef striploins.

Grass Fed				Grain Fed			
Hot Water Washed		No Water Wash		Hot water Washed		No Water Wash	
Spray Chilled	Dry Chilled	Spray Chilled	Dry Chilled	Spray Chilled	Dry Chilled	Spray Chilled	Dry chilled
Treatment 1<br>N=25	Treatment 2<br>N=25	Treatment 3<br>N=25	Treatment 4<br>N=25	Treatment 5<br>N=25	Treatment 6<br>N=25	Treatment 7<br>N=25	Treatment 8<br>N=25
